# Enhanced Thermal Stability and Synergistic Effects of Magnesium and Iron Borate Composites against Pathogenic Bacteria

**DOI:** 10.1155/2022/3605054

**Published:** 2022-11-14

**Authors:** Pervaiz Ahmad, Mayeen Uddin Khandaker, Abdulhameed Khan, Fida Rehman, Salah Ud Din, Hazrat Ali, Muhammad Imtiaz Khan, Nawshad Muhammad, Nasar Ahmed, Zahoor Ullah, Ghulamullah Khan, Sirajul Haq, Talha Bin Emran, Rohit Sharma, I. M. Ashraf

**Affiliations:** ^1^Department of Physics, University of Azad Jammu and Kashmir, 13100 Muzaffarabad, Pakistan; ^2^Center for Applied Physics and Radiation Technologies, School of Engineering and Technology, Sunway University, Bandar Sunway, 47500 Selangor, Malaysia; ^3^Department of General Educational Development, Faculty of Science and Information Technology, Daffodil International University, DIU Rd, Dhaka 1341, Bangladesh; ^4^Department of Biotechnology, University of Azad Jammu and Kashmir, 13100 Muzaffarabad, Pakistan; ^5^Department of Physics, Khushal Khan Khattak University, 27200 Karak, Khyber Pakhtunkhwa, Pakistan; ^6^Department of Chemistry, University of Azad Jammu and Kashmir, 13100 Muzaffarabad, Pakistan; ^7^Department of Physics, Abbottabad University of Science and Technology, Havelian, Khyber Pakhtunkhwa, Pakistan; ^8^Department of Dental Materials, Institute of Basic Medical Sciences, Khyber Medical University Peshawar, Khyber Pakhtunkhwa 25100, Pakistan; ^9^Department of Chemistry, Takatu Campus, Balochistan University of IT, Engineering and Management Sciences (BUITEMS), Quetta 87100, Pakistan; ^10^Department of Engineering and Architecture, Takatu Campus, Balochistan University of IT, Engineering and Management Sciences (BUITEMS), Quetta 87100, Pakistan; ^11^Department of Pharmacy, BGC Trust University Bangladesh, Chittagong 4381, Bangladesh; ^12^Department of Pharmacy, Faculty of Allied Health Sciences, Daffodil International University, Dhaka 1207, Bangladesh; ^13^Department of Rasa Shastra and Bhaishajya Kalpana, Faculty of Ayurveda, Institute of Medical Sciences, Banaras Hindu University, Varanasi, 221005 Uttar Pradesh, India; ^14^Physics Department, Faculty of Science, King Khalid University, Abha 9004, Saudi Arabia; ^15^Physics Department, Faculty of Science, Aswan University, Aswan, Egypt

## Abstract

A simple process based on the dual roles of both magnesium oxide (MgO) and iron oxide (FeO) with boron (B) as precursors and catalysts has been developed for the synthesis of borate composites of magnesium and iron (Mg_2_B_2_O_5_-Fe_3_BO_6_) at 1200°C. The as-synthesized composites can be a single material with the improved and collective properties of both iron borates (Fe_3_BO_6_) and magnesium borates (Mg_2_B_2_O_5_). At higher temperatures, the synthesized Mg_2_B_2_O_5_-Fe_3_BO_6_ composite is found thermally more stable than the single borates of both magnesium and iron. Similarly, the synthesized composites are found to prevent the growth of both gram-positive (*Staphylococcus aureus*) and gram-negative (*Escherichia coli*) pathogenic bacteria on all the tested concentrations. Moreover, the inhibitory effect of the synthesized composite increases with an increase in concentration and is more pronounced against *S. aureus* as compared to *E. coli*.

## 1. Introduction

Commercially available boron-based compounds, especially borates, are very important due to their ultimate uses in a wide range of potential applications [1]. Magnesium borates (like Mg_2_B_2_O_5_) and iron (Fe-III) borates (like Fe_3_BO_6_) are the two well-known borates in the borate family.

Mg_2_B_2_O_5_ is a magnesium borate with excellent thermal stability. Being thermodynamically stable, it has great potential to be used in making devices for working in a higher temperature environment. Besides, Mg_2_B_2_O_5_ has a great tensile strength (mechanical properties) and excellent thermoluminescent properties. It is a remarkable functional material [2], with excellent anticorrosion and antiwear behavior [3, 4]. Mg_2_B_2_O_5_ has potential applications as catalysts (for hydrocarbon conversion) and as a wide band gap semiconductor. It is used as reinforcing elements for aluminum/magnesium alloy matrix and plastics. Magnesium borates have the characteristics of thermoluminescent materials; therefore, Mg_2_B_2_O_5_ can be used in fluorescent discharge lamps and X-ray screens [5].

Like Mg_2_B_2_O_5_, Fe_3_BO_6_ (called Fe-III borates) is also a well-known borate in the iron borate family [6]. The main uses of these materials are found as electrodes in lithium-ion batteries. These electrodes (made from Fe_3_BO_6_) are used as power sources in electric and hybrid vehicles. Similarly, Fe_3_BO_6_ is used in biological probes and gas sensors [7]. Besides, Fe_3_BO_6_ can be used to work as a weak ferromagnetic switch due to its unique spin reorientation [7].

Borate compounds have excellent biocompatibility and can, therefore, be used for a series of potential applications in different biomedical fields. Likewise, borates are environmentally friendly and have a lot of potential applications in modern technology [7, 8]. The combination of borates with different bioactive materials can offer great potential in their usage in biomedical applications to avert infections. Besides borates, boron-doped silver-copper alloy nanoparticles are also very effective in eradicating *S. aureus* infection from bone cells [9].

Both of these borates (Mg_2_B_2_O_5_ and Fe_3_BO_6_) from magnesium and iron families have excellent properties for different applications in the advanced technologies. As a result, different types of structures (both in bulk and nano) of the above borates have been reported by different researchers in the literature. These included nanowires, nanorods, and whiskers. Most of the nanostructures of Mg_2_B_2_O_5_ have been obtained via chemical vapor deposition (CVD) [10], solvothermal [11], catalyst-free method [12], and solid-state synthesis, respectively [5]. However, most of the reported techniques for Mg_2_B_2_O_5_ synthesis were found to base on multisteps consisting of gas or liquid phase processes. At the same time, the yield was low and hence an economical method has been sought for the production of Mg_2_B_2_O_5_ [5]. Likewise, different nanostructures of Fe_3_BO_6_ have also been reported by solid-state reaction [13], solution-phase reaction [14, 15], hydrothermal method, etc. [16]. Again, the overall reported techniques are not only difficult to follow, time-consuming, or lengthy but also reported to have some of the as-used precursors or their compounds in the final product as impurities.

Regardless of the pros and cons of the reported techniques, Mg_2_B_2_O_5_ and Fe_3_BO_6_ are synthesized in various sizes, shapes, or morphologies. Each of the borate synthesis techniques either talks about Mg_2_B_2_O_5_ (or other magnesium borates) or Fe_3_BO_6_ (or other iron borates) in both bulk and nano for their potential applications in modern technology. However, none of the techniques so far have ever been developed to work on the synthesis of both the products as a composite. At the same time, if a technique is developed for the synthesis of borate composites (Mg_2_B_2_O_5_ and Fe_3_BO_6_), one has to make sure not only about the quality but also about the quantity of the final product. Quality and quantity are the two main parameters that need to be judged for any synthesized product to be used for its potential application. Along with the quality and quantity, the size and crystallinity of the materials also significantly change the properties to a great extent [17].

Here, we report a unique and facile technique for the synthesis of highly crystalline Mg_2_B_2_O_5_ and Fe_3_BO_6_ composites from carbon-free precursors in a growth duration of one hour at 1200°C in a graphite crucible. The as-synthesized crystalline composites of Mg_2_B_2_O_5_ and Fe_3_BO_6_ are sought to have excellent properties for their potential applications in the field of biomedical particularly in the inhibition of pathogenic bacteria. Therefore, these composites have successfully been used via the agar well diffusion method to test their antibacterial activity against both gram-positive and gram-negative pathogenic bacteria.

## 2. Experimental Details

### 2.1. Synthesis Procedure

Highly crystalline Mg_2_B_2_O_5_-Fe_3_BO_6_ composites are synthesized from a powder mixture of boron (B), iron oxide (FeO), and magnesium oxide (MgO). All the precursors were of analytical grade with a high purity of 99.999%, purchased from the Sigma-Aldrich, USA. At first, one gram powder of boron is taken in a graphite crucible. Afterward, 0.5 gram Mg powder is added to the boron powder in the crucible and homogeneously stirred for a few minutes. Consequently, 0.5 gram of FeO is also added to the crucible and properly mixed with (the mixture of) boron and MgO. The top of the crucible (open-side) is carefully covered by its graphite cap. The covered (closed) crucible (with precursors) is then placed inside the furnace for heating up to 1200°C in the argon atmosphere (where it has been kept for one hour). Afterward, the set-up is slowly brought to room temperature. The synthesized sample is carefully collected from the furnace at room temperature and characterized by different characterization apparatuses to observe its composition, phase, structure, and morphology.

### 2.2. Antibacterial Assay

The agar well diffusion method was used to test the antibacterial activity of Mg_2_B_2_O_5_-Fe_3_BO_6_ composites, against both gram-positive and gram-negative pathogenic bacteria [18]. The overnight grown culture of *E. coli* and *S. aureus* was used to assess the antibacterial activity of Mg_2_B_2_O_5_-Fe_3_BO_6_ composites. Bacterial suspensions of *E. coli* and *S. aureus* of 1.5 × 10^8^ CFU/ml concentration were inoculated on Muller-Hinton agar plates and spread uniformly via a sterile cotton swab. The same bacterial concentrations and culture medium were used for all experiments. Wells were made via a sterile polystyrene tip (4 mm). Different concentrations of Mg_2_B_2_O_5_-Fe_3_BO_6_ (20, 40, 60, 80, and 100 mg/ml) were freshly dissolved in DMSO (dimethyl sulfoxide) and added to separate wells. The plates were incubated for 24 hours at 37°C. The antibacterial activity was determined by measuring the diameter of the zone of inhibition (ZOI) produced by Mg_2_B_2_O_5_-Fe_3_BO_6_ around each well. Antibiotic clindamycin phosphate was used as a positive control to compare the antibacterial activity of Mg_2_B_2_O_5_-Fe_3_BO_6_.

## 3. Results and Discussion

The mixture of boron, FeO, and MgO was found to be an effective precursor in the synthesis of high yield boron nitride nanotubes [19–21]. After being through the initial stages with the catalytic activity of Mg and Fe with the boron, the mixture finally reacts with ammonia at higher temperatures and results in the synthesis of BNNTs. The current work is somehow similar to the previous work regarding the choice of precursors and different with respect to the use of reactive gases and experimental setup. Unlike previous, the precursors are cap-closed in the crucible. To avoid any possible contamination (as the cap of the crucible is not sealed closed) from the surrounding, argon flow is maintained throughout the whole process. No reactive gas is used. Simply, the reactants are heated up to 1200°C and kept for one hour. The following chemical reaction occurs at higher temperatures:

(1) $$\begin{array}{c} 3\text{B}+2\text{MgO}+3\text{FeO}+3{\text{O}}_{2}⟶{\text{Fe}}_{3}{\text{BO}}_{6}+{\text{Mg}}_{2}{\text{B}}_{2}{\text{O}}_{5} \end{array}$$

As a result of these reactions, highly crystalline composites of Fe_3_BO_6_ and Mg_2_B_2_O_5_ are formed at a higher temperature of 1200°C. Here, it is worth mentioning that boron has a high melting point. In the amorphous form, it melts at around 2300°C, whereas in the form of *α*-rhombohedral crystals, it melts at 2180°C [22]. Thus, based on the desires for the final product and choice of the reaction, some suitable catalysts are used to not only melt boron at an affordable temperature but also easily recovered it if not needed in the final product. Luckily, MgO and FeO have already been found to soften boron at relatively low temperatures [23]. Beside, Mg, Fe, and O are also needed as elemental contents in the required composites. Therefore, these two metal oxides are chosen to work both, initially as catalysts (soften boron to melt at a relatively lower temperature) and finally as reactants with boron to form Mg_2_B_2_O_5_-Fe_3_BO_6_ composites.

**Figure 1:**
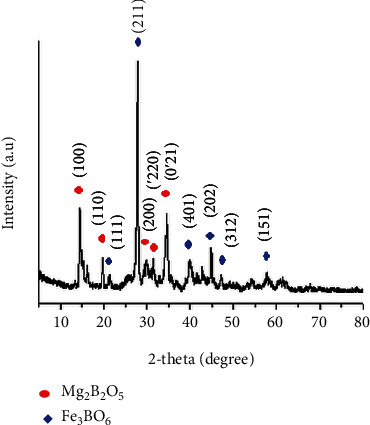
XRD pattern of the as-synthesized Mg_2_B_2_O_5_-Fe_3_BO_6_ composites.

The synthesized Mg_2_B_2_O_5_-Fe_3_BO_6_ composites are characterized by an X-ray diffractometer (XRD) to check their crystalline nature, composition, and phase. The resulted XRD pattern is displayed in Figure 1. There are several peaks in the displayed XRD pattern, which points toward the highly crystalline nature of the synthesized composites.

The peak position corresponds to the formation of different compounds in the sample. There are several peaks in the spectrum (at different 2-theta values) tagged in blue. These peaks according to the available literature correspond to (111), (211), (401), (202), (312), and (151) planes in Fe_3_BO_6_ [15, 24]. Similarly, there are several more peaks in the XRD pattern tagged in red. These red-tagged peaks in the XRD pattern stand for the planes (100), (110), (200), (′220), and (0′21) in the synthesized Mg_2_B_2_O_5_ sample according to the literature [3, 12, 25]. The existence of both the compounds (Mg_2_B_2_O_5_ and Fe_3_BO_6_) in the sample pointed toward the formation of Mg_2_B_2_O_5_-Fe_3_BO_6_ composites. The formation of these composites is confirmed in scanning electron microscopy (SEM). SEM was needed not only to check the morphology or structure of the synthesized composites but also to observe whether both the compounds are parts of the same crystals or not.

**Figure 2:**
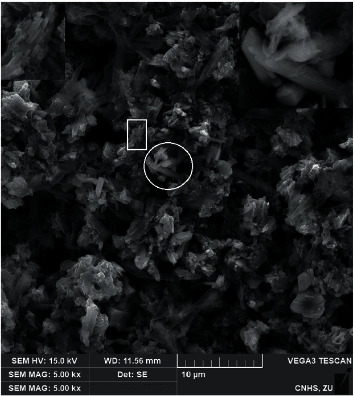
SEM micrograph of the as-synthesized Mg_2_B_2_O_5_-Fe_3_BO_6_ composites. High magnification inset on the upper right and left are the portions tagged in white circle and rectangle.

Figure 2 shows the SEM micrograph of the synthesized Mg_2_B_2_O_5_-Fe_3_BO_6_ composites. The micrograph shows randomly aligned fine crystals of Mg_2_B_2_O_5_-Fe_3_BO_6_ composites in different sizes and morphologies. None of the crystals looks identical in shape. According to the given scale in the micrograph, the sample contained crystals with a diameter in the range of 0.5–4 *μ*m. Some of the crystals are sharp and seem like randomly broken pieces of ice. Agglomerated clots of crystals can also be seen in the sample near the left-hand bottom corner of the micrograph. A few round fiber-like crystals are also found in the middle of the micrograph indicated in a white circle. The magnified view of these crystals is shown in the inset in the upper right-hand corner. The magnified view clarifies the single body structure of the composites made from Mg_2_B_2_O_5_ and Fe_3_BO_6_. Some small agglomerated structures are also indicated in the sample via a white rectangle. These structures are magnified and shown as an inset on the upper left-hand corner of the micrograph. The inset shows that the agglomerated structures are small crystals aligned in a particular pattern. It gives a clue that all of the composites start the growth in an aligned format. The growth continues until the crystal is sufficiently big enough due to the utilization of all growth species. The unavailability of the growth species causes irregular growth at higher temperatures. As a result, the crystals cannot maintain their align format and broke from their regular pattern. Thus, the synthesized Mg_2_B_2_O_5_-Fe_3_BO_6_ composites are prepared in the shown irregular sizes and morphologies.

**Figure 3:**
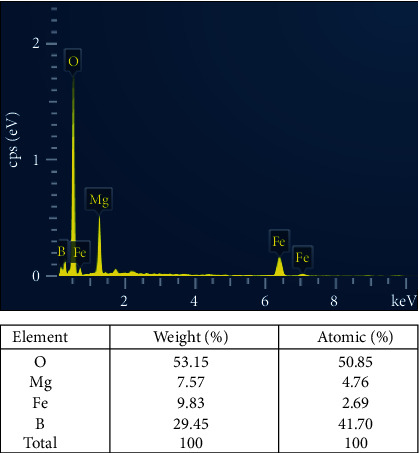
EDX spectrum showing peaks for different compositions of Mg_2_B_2_O_5_-Fe_3_BO_6_ composites. The table shows the weight and atomic percent of the elements in the synthesized composites.

Energy dispersive X-ray spectroscopy (EDX) of the synthesized Mg_2_B_2_O_5_-Fe_3_BO_6_ composite sample has been performed to verify its elemental compositions. The acquired EDX spectrum is displayed in Figure 3. The displayed spectrum has various lower and higher intensity peaks. All the reported peaks are tagged with B, O, Fe, Mg, Fe, and Fe, respectively. These tags correspond to the boron, oxygen, magnesium, and iron compositions of the synthesized sample. Their weight and atomic percentages are also given in the table below the EDX spectrum. These percentages are in good agreement with the stoichiometric calculation of the synthesized composites.

**Figure 4:**
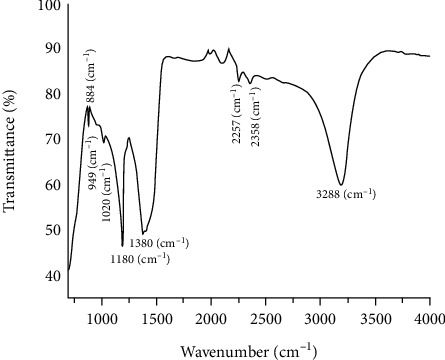
FTIR spectrum of the synthesized Mg_2_B_2_O_5_-Fe_3_BO_6_ composites.

The Mg_2_B_2_O_5_-Fe_3_BO_6_ composites are further characterized by Fourier transform infrared spectroscopy (FTIR) to analyze their composition and bonding. The obtained FTIR spectrum is shown in Figure 4. The spectrum shows two peaks at 1020 (cm^−1^) and 1180 (cm^−1^). These two peaks are linked to the asymmetric stretching of the BO_4_ group in Mg_2_B_2_O_5_ [26, 27]. The bands at 1380 (cm^−1^) are strong absorption that stands for the asymmetric stretching of the BO_3_ group. Likewise, the two small shoulder peaks found at 884 (cm^−1^) and 949 (cm^−1^) are weak absorptions due to BO_3_ group vibration in Fe_3_BO_6_ [24]. Finally, the two very weak bands at 2257 (cm^−1^) and 2358 (cm^−1^) and one very strong peak at 3288 (cm^−1^) are assigned to the stretching of O-H in hydrated borates [28, 29].

**Figure 5:**
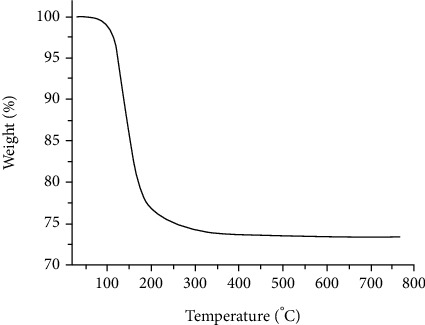
Thermogravimetric analysis curve of the as-synthesized Mg_2_B_2_O_5_-Fe_3_BO_6_ composites.

The thermal stability of the as-synthesized composites is checked in the thermogravimetric (TGA) analysis. The TGA curve of Mg_2_B_2_O_5_-Fe_3_BO_6_ composites is depicted in Figure 5. The TGA curve shows two regions of weight loss as a consequence of increasing temperature. The first weight loss is observed in the temperature range of room temperature to 200°C. This steep weight loss is attributed to the evaporation of surface water from the surface of Mg_2_B_2_O_5_-Fe_3_BO_6_ composites. The second weight loss is recorded from 200 to 300°C temperature zone. This weight loss indicates the dissociation of metal precursors used during the synthesis of borates composites. Overall, only 25 weight loss is observed up to 300°C, which verifies the thermal stability of magnesium borates and iron borate composite. Such thermal stability of both the borates is in good agreement with the literature [30, 31]. Previously, for the uncalcined powder of Mg_2_B_2_O_5_, a 42% of weight loss was recorded [4], which is quite higher than the one calculated for our synthesized composites. Likewise, the total mass loss of the synthesized composites is far less than that observed for Fe_3_BO_6_ [7]. Thus, the as-synthesized Mg_2_B_2_O_5_-Fe_3_BO_6_ composites are thermally more stable than the individual borates of iron and magnesium.

Materials have always been the main focus of researchers for different biomedical and biological applications such as antibacterial activities. In this regard, it is worth mentioning to apprise some of the excellent work done on Ag-NPs, ZnS nanoparticles, and tin-doped ZnO nanoparticles for antibacterial activities by different research groups [32–35].

Similarly, the current work is also an effort to observe the enhanced antibacterial activities of the synthesized Mg_2_B_2_O_5_-Fe_3_BO_6_ composite.

**Figure 6:**
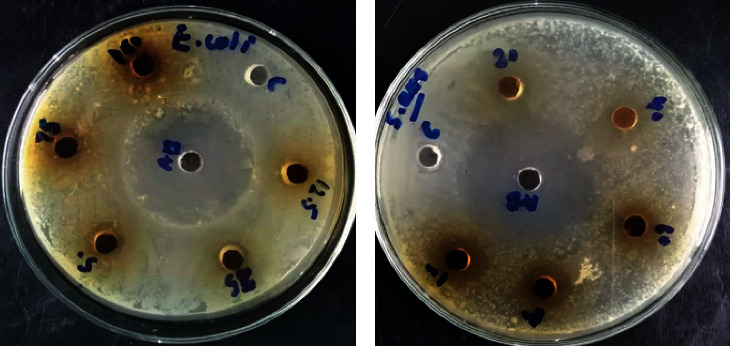
Zone of inhibition formed by different concentrations of Mg_2_B_2_O_5_-Fe_3_BO_6_ composites against *S. aureus* (a) and *E. coli* (b).

**Figure 7:**
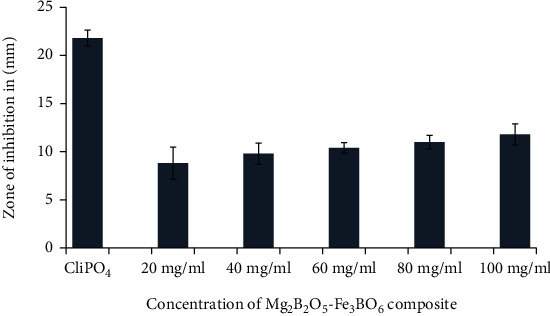
Bar chart of zone of inhibition formed by different concentrations of Mg_2_B_2_O_5_-Fe_3_BO_6_ composites against *E. coli*.

**Figure 8:**
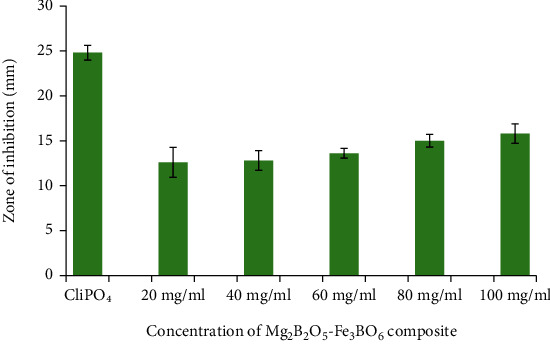
Bar chart of zone of inhibition formed by different concentrations of Mg_2_B_2_O_5_-Fe_3_BO_6_ composites against *S. aureus*.

The data obtained from the antibacterial analysis of the synthesized composite showed that Mg_2_B_2_O_5_-Fe_3_BO_6_ inhibited the growth of both gram-positive and gram-negative bacteria as shown in Figures 6(a) and 6(b). The antibacterial effect of Mg_2_B_2_O_5_-Fe_3_BO_6_ was observed against both *S. aureus* and *E. coli* on all the tested doses and found to increase with an increase in Mg_2_B_2_O_5_-Fe_3_BO_6_ composite concentration as shown in Figures 6(a) and 6(b). Against *E. coli*, the highest zone of inhibition diameter (11.8 mm) was recorded at 100 mg/ml concentration, whereas the smallest zone of inhibition diameter (8.8 mm) was observed at 20 mg/ml concentration. This can easily be seen and observed via a bar graph shown in Figure 7. Similarly, *S. aureus* showed the highest value of zone of inhibition (15.8 mm) at 100 mg/ml and the smallest zone of inhibition (12.6 mm) at 20 mg/ml (as shown in Figure 8).

The results showed that the inhibitory effect of composites was more pronounced against *S. aureus* than *E. coli* and found to form a greater zone of inhibition on all the tested doses. This is attributed to the differences in structure between gram-positive and gram-negative bacteria. The gram-positive bacteria have a thick cell wall, whereas gram-negative bacteria usually have a thin cell wall surrounded by an extra membrane.

## 4. Conclusions

Borates have excellent properties for different biological and biomedical applications including the antibacterial activity. Mg_2_B_2_O_5_ and Fe_3_BO_6_ are the two well-known magnesium and iron borates synthesized and tested for its potential applications. In view of the excellent properties of the individual borates, composites of Mg_2_B_2_O_5_ and Fe_3_BO_6_ were synthesized via a simple and easy to follow technique. The synthesized material composites of Mg_2_B_2_O_5_ and Fe_3_BO_6_ were found to have far better thermal stability than their primary compounds. The data obtained from the antibacterial study showed that the as-synthesized composites of Mg_2_B_2_O_5_-Fe_3_BO_6_ inhibited the growth of both gram-positive and gram-negative bacteria. The inhibitory effect of composites was more pronounced against *S. aureus* as compared to *E. coli* and form a greater zone of inhibition on all the tested doses. This work also revealed a potential role of boron-based composites in the inhibition of pathogenic bacteria and can therefore be used to control microbial infections.

## Data Availability

All the data is available within the manuscript.
